# Protein tertiary structure and the myoglobin phase diagram

**DOI:** 10.1038/s41598-019-47317-y

**Published:** 2019-07-25

**Authors:** Alexander Begun, Alexander Molochkov, Antti J. Niemi

**Affiliations:** 1 Laboratory of Physics of Living Matter, Far Eastern Federal University, 690950 Sukhanova 8, Vladivostok, Russia; 2 Nordita, Stockholm University, Roslagstullsbacken 23, SE-106 91 Stockholm, Sweden; 3 Institut Denis Poisson, CNRS UMR 7013, Parc de Grandmont, F37200 Tours, France; 4 Department of Physics, Beijing Institute of Technology, Haidian District, Beijing, 100081 People’s Republic of China

**Keywords:** Phase transitions and critical phenomena, Biomaterials - proteins, Biological physics

## Abstract

We develop an effective theory approach to investigate the phase properties of globular proteins. Instead of interactions between individual atoms or localized interaction centers, the approach builds directly on the tertiary structure of a protein. As an example we construct the phase diagram of (apo)myoglobin with temperature (*T*) and acidity (*pH*) as the thermodynamical variables. We describe how myoglobin unfolds from the native folded state to a random coil when temperature and acidity increase. We confirm the presence of two molten globule folding intermediates, and we predict an abrupt transition between the two when acidity changes. When temperature further increases we find that the abrupt transition line between the two molten globule states terminates at a tricritical point, where the helical structures fade away. Our results also suggest that the ligand entry and exit is driven by large scale collective motions that destabilize the myoglobin F-helix.

## Introduction

In the description of a complex system such as a protein, it is often impractical, if not impossible, to accurately model physical phenomena with a “fundamental” level precision. For example, practical chemistry, including even precision first-principles quantum chemistry, is never concerned with the detailed structure of the atomic nucleus. Instead, one focuses on a few key variables and constructs an effective theory for those. In many circumstances and in particular when the system admits either symmetries or a separation of scales, the reduced set of variables can then be treated on its own right.

In the case of proteins, the great success of structural classification schemes such as SCOP^1^ and CATH^2^ and many others^3^, demonstrates that folded proteins are built in a modular fashion, and from a relatively small number of components that are made of several amino acids. Here we exploit this modularity of protein structures to construct the phase diagram of globular proteins, with temperature (*T*) and acidity (*pH*) as the thermodynamical variables. The methodology that we develop is very general and applicable to most globular proteins^4^, even though we here develop it using myoglobin (Mg)^5–7^ as a concrete example: Our approach is based on the Landau-Ginsburg-Wilson (LGW) paradigm^8,9^ which is a systematic way to construct effective theories that model the properties of different phases in a material system, and transitions between them. Instead of individual atoms or other highly localized interaction centers and their mutual interactions, our effective theory description employs the entire tertiary structure of a protein as the fundamental constituent: We describe the protein backbone as a *single multi-soliton*^10,11^. This multi-soliton acts as an attractor in the energy landscape, it is a minimum free energy state towards which other conformations become funneled. Indeed, a multi-soliton solution to a non-linear difference (differential) equation is the paradigm structural self-organizing principle in many physical scenarios. Here it emerges as a stable solution to a variational equation that we obtain from the LGW free energy, and it governs the mutual interactions between the individual solitons that model the super-secondary structures (helix-loop-helix *etc*.) of the protein.

The advantage of the LGW formalism in combination with the soliton-concept is computational efficiency, over any other approach to protein dynamics that we are aware of; the method enables us to perform numerical simulations and analyses with very high efficiency. Similar approaches have proven highly successful in many complex scenarios with extended filament-like objects, from the description of interacting superconducting vortex lines to complex knotted fluxtubes^12,13^. Indeed, the evaluation of a *T*−*pH* phase diagram of any protein using *e.g*. molecular dynamics would be unthinkable, with presently available computers.

Myoglobin is a stable, relatively simple globular protein that is the paradigm example in protein folding and unfolding studies^14–30^. It plays an important role in biological processes such as electron transfer, oxygen delivery, catalysis and signaling. In particular, myoglobin can bind small non-polar ligands such as O_2_, CO, and NO in its interior, where they become attached to the iron atom of the heme. The native folded state (N) of myoglobin is very compact, and supports eight *α*-helices (labelled A to H). Since there is no apparent static channel for the ligands to enter and exit, myoglobin must undergo conformational deformations for the ligands to pass^5–7,31–36^. These deformations are regulated by changes in physiological conditions in particular by variations in temperature and/or acidity. Several experiments have been performed to investigate (un)folding pattern of myoglobin as a function of *pH*, mostly at room temperature. These experiments reveal that the (un)folding proceeds reversibly and sequentially, according to a four-state scheme: At low *pH* values near or below around *pH*~2 the structure resembles a random coil (U). In the vicinity of the regime ${\rm{2}}\lesssim pH\lesssim 4$ two folding intermediates I_*a*_ and I_*b*_ can be found^18,19^, both akin a molten globule^37,38^ with a structure that changes with varying *pH*. When *pH* reaches values that are above 4–4.5 (apo)Mg starts entering its native folded state. Overall, the transitions seems to proceed according to the scheme N ↔ I_*b*_ ↔ I_*a*_ ↔ U as the acidity changes^18^. Variable-*T* experiments are less common, but the results are quite similar^27–29^: At very low temperatures and near neutral *pH*, the structure of Mg is in the folded native state N; the F-helix of apoMg is disordered, even at relatively low temperatures. Below *T*~340 K and close to neutral *pH* the structure remains in the vicinity of its native state. When *T* increases further Mg becomes a molten globule, and when *T* becomes even higher the helical content starts to decrease: The first to unfold is helix F followed by helices B,C,D and E. Then the helices A, G and H loose their stability. At very high temperatures the structure resembles a random coil.

## Methods

Our effective theory approach describes the (virtual) C*α* protein backbone in terms of the (virtual) bond (*θ*) and torsion (*ϕ*) angles. To evaluate these coordinates, we frame the C*α* backbone by the mutually orthonormal backbone tangent (**t**_*i*_), binormal (**b**_*i*_) and normal (**n**_*i*_) vectors

1 $${{\bf{t}}}_{i}=\frac{{{\bf{r}}}_{i+1}-{{\bf{r}}}_{i}}{|{{\bf{r}}}_{i+1}-{{\bf{r}}}_{i}|}\,\& \,{{\bf{b}}}_{i}=\frac{{{\bf{t}}}_{i-1}\times {{\bf{t}}}_{i}}{|{{\bf{t}}}_{i-1}\times {{\bf{t}}}_{i}|}\,\& \,{{\bf{n}}}_{i}={{\bf{b}}}_{i}\times {{\bf{t}}}_{i}$$

where **r**_*i*_ (*i* = 1,..., *n*) are the C*α* coordinates. These vectors are subject to the discrete (Frenet) equation^39^

2 $$(\begin{array}{c}{{\bf{n}}}_{i+1}\\ {{\bf{b}}}_{i+1}\\ {{\bf{t}}}_{i+1}\end{array})=\exp \{\,-\,{\theta }_{i}{T}^{2}\}\exp \{\,-\,{\phi }_{i}{T}^{3}\}(\begin{array}{c}{{\bf{n}}}_{i}\\ {{\bf{b}}}_{i}\\ {{\bf{t}}}_{i}\end{array})$$

Here *T*^2^ and *T*^3^ generate three dimensional rotations, with (*T*^*i*^)_*jk*_ = *ε*_*ijk*_. From (1), (2) we can determine (*θ*_*i*_, *ϕ*_*i*_) in terms of the C*α* coordinates **r**_*i*_. Conversely, when *θ*_*i*_ and *ϕ*_*i*_ are all known we can reconstruct the **r**_*i*_ by solving (2) (for details see Supplementary Material) when we assume that the distance between neighboring C*α* atoms remains close to the average value ~3.8 Å: A good quality all-atom approximation of the entire heavy atom structure of a protein can always be reconstructed from the knowledge of the (*θ*, *ϕ*) coordinates^3,40–43^. In particular, we may employ (*θ*_*i*_, *ϕ*_*i*_) as the variables in a free energy that models the protein backbone.

Previously, a number of effective energy functions for the C*α* backbone have been constructed using the coordinates (*θ*_*i*_, *ϕ*_*i*_). Familiar examples include the fully flexible chain model and its extensions^44–46^, that are widely used in studies of biological macromolecules and other filament-like objects.

Here we introduce a free energy description that is designed to model folded proteins and their properties at the level of the tertiary structures^39,47–51^. The structure of the energy landscape is determined by the following free energy (for details see Supplementary Material)

3 $${\mathcal{F}}=\mathop{\sum }\limits_{i=1}^{n}\,\{\,-\,2{\theta }_{i+1}{\theta }_{i}+2{\theta }_{i}^{2}+\lambda \,{({\theta }_{i}^{2}-{m}^{2})}^{2}+\frac{d}{2}{\theta }_{i}^{2}{\phi }_{i}^{2}\}+\mathop{\sum }\limits_{i=1}^{n}\,\{\,-\,b\,{\theta }_{i}^{2}{\phi }_{i}-a\,{\phi }_{i}+\frac{c}{2}{\phi }_{i}^{2}\}+\sum _{i,j}\,V({{\bf{r}}}_{i}-{{\bf{r}}}_{j})$$

In the first sum of (3) we recognize the structure of the energy function of the discretized non-linear Schrödinger (DNLS) equation in the Hasimoto representation^10^. The second sum of (3) then extends the DNLS energy function so that it model folded proteins: The first two terms in the second sum are both among the conserved charges in the DNLS hierarchy, they are called the momentum and the helicity respectively. Both of these terms are odd in torsion angles, thus they break parity which makes the backbone (right-handed) chiral. The third term of the second sum is the Proca mass, together with the second term in the first sum it comprises the original Kirchhoff energy of an elastic rod^45^. Finally, the last term is a hard-core Pauli repulsion with a step-wise profile, it ensures that the distance between any two C*α* atoms is at least 3.8 Å (for detailed analysis of *V*(**r**) see^51^).

Note that there is no need to introduce any long distance contribution to *V*(**r**). The long distance interactions are already accounted for by the properties of the solution to the extended DNLS equation: The DNLS equation is the prototype integrable difference equation that supports *solitons* as its classical solutions. Solitons are the paradigm examples of extended self-organized objects in a physical system^11^. For appropriate parameter values the DNLS free energy (3) models the entire tertiary structure of a given folded protein backbone, as a single stable minimum energy multi-soliton solution to the variational equations

4 $$\frac{\delta  {\mathcal F} }{\delta {\theta }_{i}}=2(2{\theta }_{i}-{\theta }_{i+1}-{\theta }_{i-1})+4\lambda ({\theta }_{i}^{2}-{m}^{2}){\theta }_{i}+(d{\phi }_{i}^{2}-2b{\phi }_{i}){\theta }_{i}=0$$

5 $$\frac{\delta  {\mathcal F} }{\delta {\phi }_{i}}=(d{\theta }_{i}^{2}+c){\phi }_{i}-b{\theta }_{i}^{2}-a=0$$

The multi-soliton profile then describes the various super-secondary structures such as helix-loop-helix (regular-loop-regular) as mutually interacting individual solitons^52,53^. Over a single soliton profile the parameter values in (3) are uniform, and since a soliton extends over several amino acids the number of parameters is generically much smaller than the number of amino acids. In the case of a myoglobin, we use the Protein Data Bank (PDB) structure 1ABS (sperm whale)^33^ as a decoy to construct the parameters. This structure has been measured at the very low liquid helium temperature value of around 20 Kelvin and as a consequence the thermal B-factors are very small. We identify ten individual DNLS solitons profiles along the 154 residue 1ABS backbone that become combined into a single multi-soliton solution of the DNLS equations (4), (5) with ~0.8 Å C*α* root-mean-square-distance (RMSD) precision^47^ (see also Supplementary Material).

We construct the *T*-*pH* phase diagram by computing the grand canonical ensemble of statistical physics, and evaluate the observables ${\mathscr{O}}(\theta ,\phi )$ by averaging them over all possible tertiary structures, weighted by the grand canonical distribution with free energy (3):

6 $$ < {\mathscr{O}}(\theta ,\phi ){ > }_{\beta ,\mu }=\frac{1}{{\mathscr{Z}}}Tr\{{\mathscr{O}}(\theta ,\phi ){e}^{-\beta ( {\mathcal F} -\mu N)}\}$$

Here ${\mathscr{Z}}$ is a normalization factor, *β* is the inverse temperature factor and *μN* is a chemical potential contribution to be specified. Since the trace extends over all possible tertiary structures, the entropy contribution relates to the number of all possible tertiary structures. We evaluate (6) numerically, using the Glauber algorithm with acceptance ratio determined by the probability distribution^51,54,55^

7 $${\mathscr{P}}=\frac{{e}^{-\beta \Delta ( {\mathcal F} -\mu N)}}{1+{e}^{-\beta \Delta ( {\mathcal F} -\mu N)}}$$

Here $\Delta ( {\mathcal F} -\mu N)$ is the variation of ${\mathcal F} -\mu N$ between consecutive Monte Carlo steps. We note that Glauber algorithm models pure relaxation dynamics, and for simple systems it reproduces Arrhenius law. At the same time it has been found that small proteins fold according to Arrhenius law^56^. We also note that the (inverse of the) Glauber temperature factor *β* does not coincide with physical temperature factor *kT* where *k* is the Boltzmann constant and *T* is measured in Kelvin’s, instead the relation is determined by renormalisation group techniques^47,57^.

We determine the chemical potential contribution in (6), by recalling the Henderson-Hasselbalch equation^58^ that relates the concentrations of protonated and non-protonated amino acids to the difference between *pH* and acid dissociation constant *pK*_*a*_. On the other hand, Gibbs free energy is commonly taken to vary with acidity as follows,

8 $$\Delta G=RT\,\mathrm{ln}(10)\sum _{a}\,(pH-p{K}_{a})=RT\,\sum _{a}\,\mathrm{ln}(\frac{1-{{\mathscr{P}}}_{H}^{a}}{{{\mathscr{P}}}_{H}^{a}})$$

where ${{\mathscr{P}}}_{H}^{a}$ is the protonation probability of a particular amino acid. Histidine with *pK*_*a*_~6.0 is the only amino acid in the genetic code that has strong reactivity to *pH* variations in the physiologically important range from *pH*~8 down to *pH*~4. For lower *pH* both glutamic acid (*pK*_*a*_~4.2) and aspartic acid (*pK*_*a*_~3.9) need to be accounted for. For simplicity, here we only aim to model the phase diagram for *pH* above ~4 and up to neutral value so that we only need to account to the contribution of the ${N}_{H}^{his}=12$ histidines in 1ABS. Then

9 $${{\mathscr{P}}}_{H}^{his}=\frac{{e}^{-\Delta G/RT}}{1+{e}^{-\Delta G/RT}}$$

and to leading order

$$\Delta G\approx RT\,\mathrm{ln}(10)(pH-p{K}_{a}){N}_{H}^{his}\frac{{e}^{-\mathrm{ln}(10)(pH-p{K}_{a})}}{1+{e}^{-\mathrm{ln}(10)(pH-p{K}_{a})}}$$

We recognize in (9) the format of the Glauber transition probability (7). Moreover, since the DNLS hierarchy admits a *unique* conserved number operator *N*~*θ*^2^ ^10^ we propose that in the LGW approach

10 $$G \sim  {\mathcal F} -\mu N= {\mathcal F} -\mu \sum _{i\in his}\,{\theta }_{i}^{2}$$

where the summation extends over the histidines of 1ABS. As a consequence, to leading order in the LGW approximation *μ* depends linearly on *pH*. We also note that for 1ABS *pH* = 9.0. Accordingly we normalize *μ* = 0 at that value, to ensure that the ensuing multi-soliton profile models the 1ABS backbone.

As order parameters *a.k.a*. reaction coordinates we use the radius of gyration *R*_*g*_ and the *α*-helical content ${{\mathscr{Q}}}_{\alpha }$. We compute their (*T*, *μ*) dependence numerically from (6). We take a C*α* atom to be in an *α*-helical posture when for (*θ*_*i*_, *ϕ*_*i*_) both |*θ*_*i*_−*θ*_0_| ≤ 0.14 (rad) and |*ϕ*_*i*_−*ϕ*_0_| < 0.3 (rad) where *θ*_0_ = 1.55 and *ϕ*_0_ = 0.9 are the PDB average values of the *α*-helical bond and torsion angle. The ${{\mathscr{Q}}}_{\alpha }$ counts the relative number of residues in *α*-helical posture as a function of *T* and *pH*; most PDB myoglobins have a ${{\mathscr{Q}}}_{\alpha }$ value 72–78%, and for 1ABS ${{\mathscr{Q}}}_{\alpha }=72 \%$.

We have simulated 5.000 independent heating and cooling (unfolding and folding) trajectories using the Glauber algorithm, obtained by varying the (inverse) temperature factor *β*; the trajectories are equally distributed between 50 values of *μ* ∈ [0, 0.05]. Along each trajectory we first increase temperature (*i.e*. decrease *β*) at an adiabatically slow rate, so that the system remains very close to a thermal equilibrium for all *β*. The value of *β* is also kept at its high temperature value for a large number of simulation steps, for full thermalization. Finally, the system is brought back to the low temperature value, by reversal of the heating procedure: We have been extremely careful to always thermalize the ensemble before we evaluate any observable^51^.

## Results

**Figure 1:**
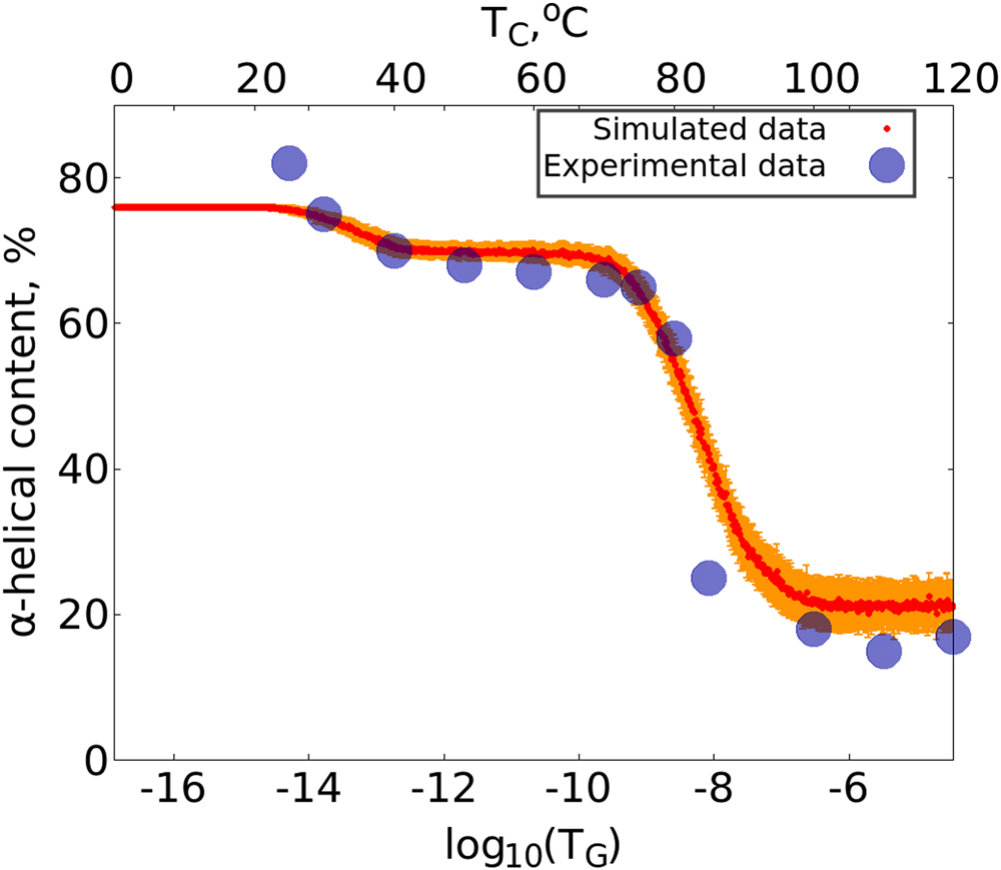
Comparison between simulated (sperm whale) value ${{\mathscr{Q}}}_{\alpha }$ that counts the relative number of residues in *α*-helical posture, and experimentally determined (horse heart) *α*-helical content during thermal denaturation. Experimental data is adapted from^28^.

**Figure 2:**
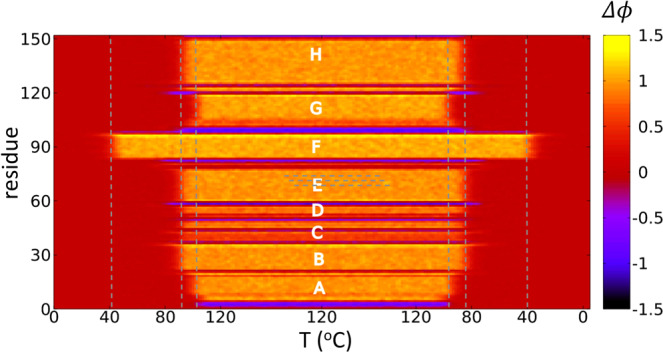
(Dis)ordering temperatures for the eight helices A-H at *μ* = 0, in terms of deviations in torsion angles from their *α*-helical values. Note that helix-F becomes disordered at relatively low temperature.

**Figure 3:**
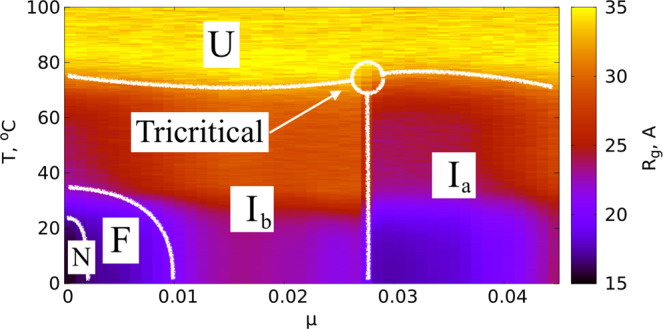
The *R*_*g*_ phase diagram: Collapsed native state (N), (dis)ordering of F-helix (F), two molten globules (I_*a*_ and I_*b*_) and random coil (U) phase are identified. Note the presence of an apparent tricritical point, when the transition line between I_*a*_ and I_*b*_ terminates in U.

**Figure 4:**
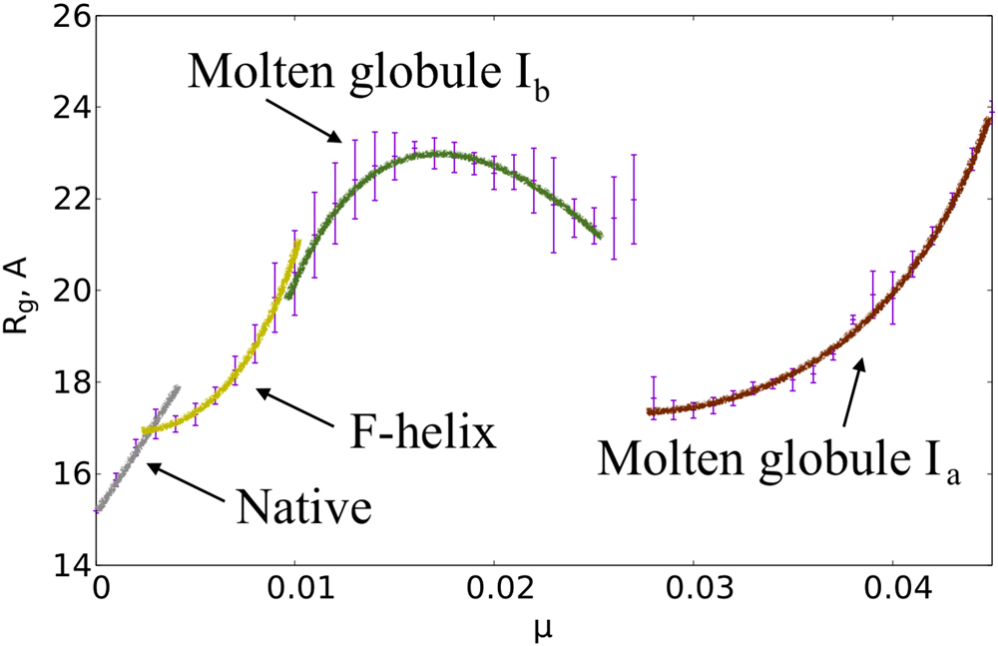
The evolution of *R*_*g*_ at temperature close to *T* = 0 °C. The native state, the state with disordered F-helix and the two molten globule states I_*a*_ and I_*b*_ are all identifiable.

**Figure 5:**
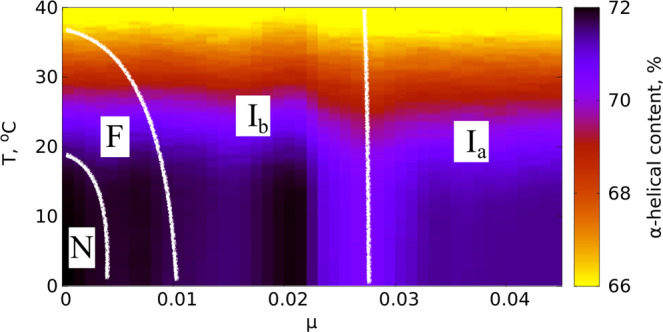
The phase diagram in terms of the observable ${{\mathscr{Q}}}_{\alpha }$ that counts the relative number of residues in *α*-helical posture, at low temperatures *T* < 40 °C.

In Fig. 1 we compare the *μ* = 0 temperature dependence of the observable ${{\mathscr{Q}}}_{\alpha }$ to experimentally measured *α*-helicity of (horse heart) myoglobin during thermal denaturation. The experimental data is adapted from^28^. We use this Figure to relate the Glauber temperature *T*_*G*_ to Celsius scale. Accordingly, our simulations cover the range 0 °C–120 °C at each *μ* value. The Figs 2–5 summarizes our findings:

- Fig. 2 shows the helix (dis)ordering during a heating and cooling simulation cycle, as a function of temperature at *μ* = 0 and in terms of the average value of torsion angles *ϕ*; we recall that for an *α*-helix *ϕ* ≈ 1 (rad).
  We observe that *F*-helix starts to disorder soon after *T* = 20 ^°^C and becomes fully randomized slightly above *T* = 40 °C. The next to disorder are the helices B, C, D and E; this occurs near *T* = 90 °C. This is followed by disordering of the helices H and A, and the helix G is last to disorder. Slightly above *T* = 100 °C the entire chain is fully randomized; according to Fig. 1, at these temperature values ${{\mathscr{Q}}}_{\alpha }$ also reaches its high temperature asymptotic value. All these simulation results are fully in line with experimental observations^24^.
- Fig. 3 identifies the simulated phase structure on the (*T*, *μ*) plane in terms of radius of gyration *R*_*g*_. For *μ* ≈ 0 we confirm the findings of the Figs 1 and 2: The native state (N) is a region with low temperature (*T* < 30 °C) and very small values *μ* < 0.003. Beyond this there is a region where the F-helix (dis)orders (F), it extends to around *T* ≈ 40 °C and to *μ*-values up to *μ* ≈ 0.01. When *T* and *μ* increase further, we identify a phase that we denote I_*b*_ and identify as a molten globule intermediate; the radius of gyration values are above 20 Å but below 28 Å in this region, depending on values of *T* and *μ*.
  We propose that the high sensitivity of the F-helix that we observe, when either temperature or acidity increases from their low values, controls the ligand entry and exit: The F-helix contains the proximal histidine that is connected to the heme. Thus disordering of the F-helix may expose the heme, for ligand transport.
  At *μ* ≈ 0.027 and for values *T* < 80 °C we observe a rapid transition: The radius of gyration decreases in a jump-like fashion, by around 4–6 Å depending on *T*. We interpret this to be a transition between two molten globule intermediates so that for *μ* > 0.027 we have the second molten globule I_*a*_^18,19^.
  Most notably, we observe the presence of an apparent tricritical point, in conjuction with the two molten globule states: The transition line between I_*a*_ and I_*b*_ terminates at around *T* ≈ 80 °C when both molten globules simultaneously enter the random coil phase.
- The different regions of the phase diagram in Fig. 3 can be scrutinized using the detailed *R*_*g*_ values. In Fig. 4 we show how *R*_*g*_ varies as a function of *μ*, at *T* = 0 °C. We observe a clear change in the derivate of *R*_*g*_ *w.r.t. μ* at around *μ* ≈ 0.003 and also around *μ* ≈ 0.01. These correspond to the transitions between the N and F, and between the F and I_*b*_ regions in the phase diagram on Fig. 2. Note that the *R*_*g*_ value of the molten globule I_*b*_ is very close to the experimentally reported value *R*_*g*_~23.6 Å^16,21,24^.
  We also have a jump-like (discontinuous) transition in *R*_*g*_ values at around *μ* = 0.027, between the two molten globules I_*b*_ and I_*a*_.
- Finally, in Fig. 5 we display the values of ${{\mathscr{Q}}}_{\alpha }$ that counts the relative number of residues in *α*-helical posture, for *T* < 40 °C. We observe that there is only a weak dependence on *μ*, even though we do note a slight change in the overall stability of helix-F even at relatively small *μ* values. We conclude that at low temperatures the increase of *μ* appears to have a stronger influence on loops than on helices. In particular, the ligand transport mechanism if indeed associated with instability in helix-F, appears to engage the adjacent loop structures as well.

The results in Fig. 5 are consistent with room temperature CD helicity measurements that report only minor signal variations for *pH* values above ~4.5^23^: Acidity does not have a strong effect on the hydrogen bonds that stabilize the helical structures. In the Figure we identify the transition between I_*b*_ and I_*a*_, in terms of a region with (slightly) decreased value of ${{\mathscr{Q}}}_{\alpha }$. It is also notable that right prior to the transition, there is region in I_*b*_ with an enhanced ${{\mathscr{Q}}}_{\alpha }$ values.

## Discussion

In summary, we have proposed to model protein thermodynamics directly at the tertiary level of structures, in terms of the multi-soliton solution of the DNLS equation. We have numerically evaluated the ensuing grand canonical partition function at finite temperature and chemical potential, with the latter identified by comparison with the Henderson-Hasselbalch equation. As an example we have constructed the (*T*, *pH*) phase diagram of myoglobin. All our results are in a good agreement with experimental observations. In particular, the ordering of helix stabilization and the emergence of two molten globules are qualitatively in full agreement with experimental observations. Furthermore, we observe that the F-helix with its proximal histidine, is the first to loose stability as either temperature or acidity increase from neutral values. This supports that the destabilization of the F-helix region might have a pivotal role for ligand entry and exit. We have also made predictions for future experiments, in particular we have proposed that at high temperatures near *T* = 80 °C there is an apparent tricritical point where the two molten globules come together with the random coil phase. Our results show that effective theories that model protein structure directly at the tertiary structure level, can provide a viable computational approach to investigate the phase structure of complex globular proteins.

## Supplementary information

Supplementary Dataset 1
