# Supplementary material for: Protein tertiary structure and the myoglobin phase diagram
Source: Sci Rep. 2019 Jul 25;9:10819. doi: 10.1038/s41598-019-47317-y (PMC6658483; doi:10.1038/s41598-019-47317-y)
Supplement: Supplementary file 1 — Supplementary Dataset 1 [file 41598_2019_47317_MOESM1_ESM.pdf]

SUPPLEMENTARY MATERIAL

Protein tertiary structure and the myoglobin phase diagram

Alexander Begun,[1,*] Alexander Molochkov,[1,†] and Antti J. Niemi[2,3,1,4,‡]

[1] *Laboratory of Physics of Living Matter, Far Eastern Federal University, 690950, Sukhanova 8, Vladivostok, Russia*

[2] *Nordita, Stockholm University, Roslagstullsbacken 23, SE-106 91 Stockholm, Sweden*

[3] *Institut Denis Poisson, CNRS UMR 7013, Parc de Grandmont, F37200, Tours, France*

[4] *Department of Physics, Beijing Institute of Technology, Haidian District, Beijing 100081, People's Republic of China*

Abstract

We describe how the Kirchhoff elastic rod model is generalized to the nonlinear Schrödinger equation and how the energy function used in the article is a consequence.

- Continuum Frenet equation and effect of frame rotations
- Kirchhoff elastic rod and the nonlinear Schrödinger equation
- Solitons
- Discrete Frenet equation and the discretized nonlinear Schrödinger equation
- Soliton model of myoglobin (used in the article)

* beg.alex93@gmail.com

† molochkov.alexander@gmail.com

‡ Antti.Niemi@su.se

I. CONTINUUM CURVES AND GENERALIZED KIRCHHOFF'S ELASTIC ROD

A. The Frenet Equation

The geometry of a class $\mathcal{C}^3$ differentiable curve $\mathbf{x}(s)$ in $\mathbb{R}^3$ is governed by the Frenet equation, described widely in elementary courses of differential geometry [1]. We parametrize the curve with its proper length $s \in [0, L]$ where L is the length of the curve in $\mathbb{R}^3$. We introduce the unit length tangent vector

$$\mathbf{t} \; = \; \frac{d\mathbf{x}(s)}{ds} \; \equiv \; \mathbf{x}_s \tag{1}$$

the unit length bi-normal vector

$$\mathbf{b} \; = \; \frac{\mathbf{x}_s \times \mathbf{x}_{ss}}{||\mathbf{x}_s \times \mathbf{x}_{ss}||} \tag{2}$$

and the unit length normal vector,

$$\mathbf{n} = \mathbf{b} \times \mathbf{t} \tag{3}$$

The three vectors $(\mathbf{n}, \mathbf{b}, \mathbf{t})$ define the orthonormal, right-handed Frenet frames. We can introduce this framing at every point along the curve, whenever

$$\mathbf{x}_s \times \mathbf{x}_{ss} \; \neq \; 0 \tag{4}$$

The Frenet equation transports the frames along the curve as follows,

$$\frac{d}{ds}\begin{pmatrix} \mathbf{n} \\ \mathbf{b} \\ \mathbf{t} \end{pmatrix} \; = \; \begin{pmatrix} 0 & \tau & -\kappa \\ -\tau & 0 & 0 \\ \kappa & 0 & 0 \end{pmatrix} \begin{pmatrix} \mathbf{n} \\ \mathbf{b} \\ \mathbf{t} \end{pmatrix} \tag{5}$$

Here

$$\kappa(s) \; = \; \frac{||\mathbf{x}_s \times \mathbf{x}_{ss}||}{||\mathbf{x}_s||^3} \tag{6}$$

is the curvature and

$$\tau(s) \; = \; \frac{(\mathbf{x}_s \times \mathbf{x}_{ss}) \cdot \mathbf{x}_{sss}}{||\mathbf{x}_s \times \mathbf{x}_{ss}||^2} \tag{7}$$

is the torsion. Both $\kappa(s)$ and $\tau(s)$ are extrinsic geometric quantities *i.e.* they depend only on the shape of the curve in $\mathbb{R}^3$. Conversely, if we know the curvature and torsion we can construct the curve, by first solving for $\mathbf{t}(s)$ from the Frenet equation followed by integration of (1). The solution is unique, modulo a global translation and rotation.

B. Frame rotation

We start with the observation that the normal and bi-normal vectors do not appear in (1). As a consequence a rotation around $\mathbf{t}(s)$,

$$\begin{pmatrix} \mathbf{n} \\ \mathbf{b} \end{pmatrix} \rightarrow \begin{pmatrix} \mathbf{e}_1 \\ \mathbf{e}_2 \end{pmatrix} = \begin{pmatrix} \cos\eta(s) & \sin\eta(s) \\ -\sin\eta(s) & \cos\eta(s) \end{pmatrix} \begin{pmatrix} \mathbf{n} \\ \mathbf{b} \end{pmatrix}. \tag{8}$$

has no effect on the curve. For the Frenet equation this rotation gives

$$\frac{d}{ds} \begin{pmatrix} \mathbf{e}_1 \\ \mathbf{e}_2 \\ \mathbf{t} \end{pmatrix} = \begin{pmatrix} 0 & (\tau + \partial_s \eta) & -\kappa\cos\eta \\ -(\tau + \partial_s \eta) & 0 & -\kappa\sin\eta \\ \kappa\cos\eta & \kappa\sin\eta & 0 \end{pmatrix} \begin{pmatrix} \mathbf{e}_1 \\ \mathbf{e}_2 \\ \mathbf{t} \end{pmatrix}. \tag{9}$$

The form of (9) suggests to combine the two κ dependent contributions into a single complex quantity [2–4],

$$\kappa \xrightarrow{\eta} \kappa(\cos\eta + i\sin\eta) \equiv \kappa e^{i\eta} \tag{10}$$

We may then introduce the following notations/conventions when representing curvature and torsion in arbitrary frame,

$$\begin{aligned} \kappa \rightarrow \ \kappa e^{-i\eta} &\equiv \phi \\ \tau \rightarrow \tau + \partial_s \eta &\equiv \sqrt{\tfrac{d}{2}} A_i \end{aligned} \tag{11}$$

Here d is a parameter that we introduce for future convenience; for the Frenet equations we may set $d = 2$. With these variables, (9) admits the manifestly frame covariant form:

$$\begin{aligned} (\tfrac{d}{ds} \mp i\sqrt{\tfrac{d}{2}} A)\mathbf{e}_\pm &= -\phi\mathbf{t} \\ \tfrac{d}{ds}\mathbf{t} &= \tfrac{1}{2}(\phi\,\mathbf{e}_+ + \bar{\phi}\,\mathbf{e}_-) \end{aligned} \tag{12}$$

with

$$\mathbf{e}_\pm = \mathbf{e}_1 \pm i\mathbf{e}_2 \quad \Rightarrow \quad \mathbf{e}_\pm \rightarrow e^{\pm i\eta}\mathbf{e}_\pm$$

and we remind that $\mathbf{t}$ is frame invariant.

C. The Kirchhoff elastic rod and its generalizations

The curvature and torsion are the only quantities available to construct energy functions for filamentous, inextensible elastic rods. According to Kirchhoff the energy is [5]

$$E \; = \; \int_0^L ds\{\alpha\kappa^2 + \beta\tau^2\} \tag{13}$$

where α and β are some parameters. The case $\beta = 0$ corresponds to Euler's elastica; in a biological context this defines the worm like chain (WLC) model that is commonly used to describe long and flexible linear (bio)polymers [2]

The energy function (13) describes the bending and twisting of a thin rod in the limit of very small curvature and torsion. But this energy function is not capable of describing phenomena such a supercoiling, nor structures such as helix-loop-helix that are common in case of proteins. For this we need to include higher order, non-linear contributions to (13). To do this systematically, we need a guiding principle: Note that even though framing is a necessary intermediate step to construct the curve from the knowledge of its curvature and torsion, the shape of a curve can not depend on the way how it is framed. Indeed, the Frenet equations can be presented in the frame covariant form (12). Thus, the energy function should similarly admit a frame covariant form, one that is the same independently of the framing when expressed in the frame covariant variables (ϕ, A) in (11). An example of a frame covariant energy function is [2–4],

$$H \; = \; \int_0^L ds \left\{ |(\partial_s + i\sqrt{\frac{d}{2}}A)\phi|^2 + \lambda\,(|\phi|^2 - m^2)^2 \; - \; a\,A + \frac{c}{2}A^2 \right\} \tag{14}$$

The first two terms have the functional form of the Hamiltonian that appears in the Abelian Higgs model. They remain *manifestly* intact under a frame rotation (11).

The third term, with parameter a, is the one dimensional Chern-Simons term. It breaks chirality which ensures that the curves are chiral, either right-handed or left-handed depending on the sign of parameter a. Note that under a frame rotation this terms transforms by a derivative; see (11). Thus it remains invariant when there are no end point frame rotations.

The fourth term in (14) is called the Proca mass in the context of the Abelian Higgs model. It is *not* covariant under a frame rotation but we included it for completeness since it yields the second term in (13), in Frenet frames.

D. Energy and soliton of Nonlinear Schrödinger equation

In term of the geometric curvature and torsion, the energy density of (14) translates to

$$\mathcal{H} \ = \ (\partial_s \kappa)^2 + \frac{d}{2}\kappa^2\tau^2 + \lambda\,(\kappa^2 - m^2)^2 \ - \ a\,\tau \ + \ \frac{c}{2}\,\tau^2 \tag{15}$$

We introduce the Hasimoto variable [3, 4, 6], to combine the curvature and torsion into a single frame invariant complex quantity

$$\psi(s) \ = \ \kappa(s)\exp\{i\int\limits_0^s ds'\,\tau(s')\} \ \equiv \ \phi(s)\exp\{i\sqrt{\frac{d}{2}}\int\limits_0^s ds'\,A(s')\} \tag{16}$$

In terms of (16), we find that (15) includes the following,

$$(\partial_s \kappa)^2 + e^2\kappa^2\tau^2 + \lambda\kappa^4 \ = \ \bar\psi_s\psi_s + \lambda(\bar\psi\psi)^2 \ = \ \mathcal{H}_3 \tag{17}$$

This the energy density of the standard nonlinear Schrödinger equation (NLS), the paradigm integrable model that supports solitons as classical solutions: The non-vanishing Poisson bracket of the Hasimoto variables is

$$\{\psi(s), \bar\psi(s')\} \ = \ i\delta(s - s')$$

and the following quantities are conserved densities in the sense that their Poisson brackets with $\mathcal{H}_3$ vanish [3, 6?]

$$\begin{aligned}
\mathcal{H}_{-2} &= \tau \\
\mathcal{H}_{-1} &= L \\
\mathcal{H}_1 &= \kappa^2 \quad \sim \bar\psi\psi \\
\mathcal{H}_2 &= i\kappa^2\tau \ \ \sim \bar\psi\psi_s
\end{aligned} \tag{18}$$

The energy (15) is a combination of $\mathcal{H}_{-2}$, $\mathcal{H}_1$ and $\mathcal{H}_3$, except for its last term, the Proca mass. From the perspective of the NLS hierarchy, the momentum $\mathcal{H}_2$ should also be included so that at the end we have the energy density

$$\mathcal{H} \ = \ (\partial_s \kappa)^2 + \frac{d}{2}\kappa^2\tau^2 + \lambda\,(\kappa^2 - m^2)^2 \ - \ b\kappa^2\tau \ - \ a\tau \ + \ \frac{c}{2}\tau^2 \tag{19}$$

The standard NLS equation is the paradigm equation that supports solitons [7, 8]; depending on the sign of λ the soliton is either dark ($\lambda > 0$) or bright ($\lambda < 0$). In particular, the torsion independent contribution

$$(\partial_s \kappa)^2 + \lambda\,(\kappa^2 - m^2)^2 \tag{20}$$

supports the double well *topological* soliton: When m^2 is positive and when κ can take both positive and negative values, the equation of motion

$$\partial_{ss}\kappa = 2\lambda\kappa(\kappa^2 - m^2)$$

is solved by

$$\kappa(s) \;=\; m \tanh\left[m\sqrt{\lambda}(s - s_0)\right] \tag{21}$$

The energy function (19) is quadratic in the torsion. Thus we can eliminate τ using its equation of motion,

$$\tau[\kappa] \;=\; \frac{a + b\kappa^2}{c + d\kappa^2} \;\equiv\; \frac{a}{c}\,\frac{1 + (b/a)\kappa^2}{1 + (d/c)\kappa^2} \tag{22}$$

and we obtain the following equation of motion for curvature,

$$\kappa_{ss} = V_\kappa[\kappa] \tag{23}$$

where

$$V[\kappa] \;=\; -\left(\frac{bc - ad}{d}\right)\frac{1}{c + d\kappa^2} \;-\; \left(\frac{b^2 + 8\lambda m^2}{2b}\right)\kappa^2 + \lambda\,\kappa^4 \tag{24}$$

This shares the same large-κ asymptotics, with the potential in (20). With properly chosen parameters, we expect that (23), (24) continue to support topological solitons, but we do not know their explicit profile, in terms of elementary functions.

The curve is the constructed as follows: Once we have the soliton of (23), we evaluate $\tau(s)$ from (22). We substitute the ensuing (κ, τ) profiles in the Frenet equation (5) and solve for $\mathbf{t}(s)$. We then integrate (1) to obtain the curve $\mathbf{x}(s)$ that corresponds to the soliton. A generic soliton curve looks like a helix-loop-helix motif (more generally a regular secondary structure - a loop - a regular secondary structure), familiar from crystallographic protein structures.

II. POLYGONS AND GENERALIZED KIRCHHOFF ENERGIES

A. Discrete Frenet equation

Proteins are not alike continuous, differentiable curves. Proteins are like piecewise linear polygonal chain. Thus, to construct a generalized Kirchhoff model applicable for proteins, we need to generalise the Frenet frame formalism to the case of a polygonal, piecewise linear chain [9].

Let $\mathbf{r}_i$ with $i = 1, ..., N$ be the vertices of the chain. At each vertex we introduce the unit tangent vector

$$\mathbf{t}_i = \frac{\mathbf{r}_{i+1} - \mathbf{r}_i}{|\mathbf{r}_{i+1} - \mathbf{r}_i|} \tag{25}$$

the unit binormal vector

$$\mathbf{b}_i = \frac{\mathbf{t}_{i-1} - \mathbf{t}_i}{|\mathbf{t}_{i-1} - \mathbf{t}_i|} \tag{26}$$

and the unit normal vector

$$\mathbf{n}_i = \mathbf{b}_i \times \mathbf{t}_i \tag{27}$$

The orthonormal triplet $(\mathbf{n}_i, \mathbf{b}_i, \mathbf{t}_i)$ defines a discrete version of the Frenet frames (1)-(3) at each position $\mathbf{r}_i$ along the chain.

In lieu of the curvature and torsion, we have their discrete analogues, the bond angles and torsion angles. When we know the vertices we also know the Frenet frames and we can compute these angles: The bond angles are

$$\theta_i \;\equiv\; \theta_{i+1,i} \;=\; \arccos\left(\mathbf{t}_{i+1} \cdot \mathbf{t}_i\right) \tag{28}$$

and the torsion angles are

$$\psi_i \;\equiv\; \psi_{i+1,i} \;=\; \mathrm{sign}\{\mathbf{b}_{i-1} \times \mathbf{b}_i \cdot \mathbf{t}_i\} \cdot \arccos\left(\mathbf{b}_{i+1} \cdot \mathbf{b}_i\right) \tag{29}$$

Conversely, when the values of the bond and torsion angles are all known, we can use the discrete version of the Frenet equation (5)

$$\begin{pmatrix} \mathbf{n}_{i+1} \\ \mathbf{b}_{i+1} \\ \mathbf{t}_{i+1} \end{pmatrix} = \begin{pmatrix} \cos\theta\cos\psi & \cos\theta\sin\psi & -\sin\theta \\ -\sin\psi & \cos\psi & 0 \\ \sin\theta\cos\psi & \sin\theta\sin\psi & \cos\theta \end{pmatrix}_{i+1,i} \begin{pmatrix} \mathbf{n}_i \\ \mathbf{b}_i \\ \mathbf{t}_i \end{pmatrix} \tag{30}$$

to compute the frame at position $i + i$ from the frame at position i. Once all the frames have been constructed, the entire string is given by discrete version of (1),

$$\mathbf{r}_k = \sum_{i=0}^{k-1} |\mathbf{r}_{i+1} - \mathbf{r}_i| \cdot \mathbf{t}_i \tag{31}$$

In the case of a protein, it is sufficient to take $|\mathbf{r}_{i+1} - \mathbf{r}_i| = 3.8$Å; this is the average distance between neighboring Cα atoms. The bond oscillations are very fast, and over time intervals in the scale of microsecond the average values can be used.

In constructing the chain, without any loss of generality we may choose $\mathbf{r}_0 = 0$, make $\mathbf{t}_0$ to point into the direction of the positive z-axis, and let $\mathbf{t}_1$ lie on the y-z plane.

B. frame rotations

The vectors $\mathbf{n}_i$ and $\mathbf{b}_i$ do not appear in (31). Thus, as in the case of continuum curves, a discrete chain remains intact under frame rotations of the $(\mathbf{n}_i, \mathbf{b}_i)$ zweibein around $\mathbf{t}_i$. This local SO(2) rotation acts on the frames as follows [9]

$$\begin{pmatrix} \mathbf{n} \\ \mathbf{b} \\ \mathbf{t} \end{pmatrix}_i \to e^{\Delta_i T^3} \begin{pmatrix} \mathbf{n} \\ \mathbf{b} \\ \mathbf{t} \end{pmatrix}_i = \begin{pmatrix} \cos\Delta_i & \sin\Delta_i & 0 \\ -\sin\Delta_i & \cos\Delta_i & 0 \\ 0 & 0 & 1 \end{pmatrix} \begin{pmatrix} \mathbf{n} \\ \mathbf{b} \\ \mathbf{t} \end{pmatrix}_i \tag{32}$$

Here Δ_i is the rotation angle at vertex i and T^3 is one of the SO(3) generators

$$T^1 = \begin{pmatrix} 0 & 0 & 0 \\ 0 & 0 & -1 \\ 0 & 1 & 0 \end{pmatrix} \quad T^2 = \begin{pmatrix} 0 & 0 & 1 \\ 0 & 0 & 0 \\ -1 & 0 & 0 \end{pmatrix} \quad T^3 = \begin{pmatrix} 0 & -1 & 0 \\ 1 & 0 & 0 \\ 0 & 0 & 0 \end{pmatrix}$$

that satisfy the Lie algebra

$$[T^a, T^b] = \epsilon^{abc} T^c$$

Using these matrices we can write the effect of frame rotation on the bond and torsion angles as follows

$$\theta_i \, T^2 \;\to\; e^{\Delta_i T^3} (\theta_i T^2) \, e^{-\Delta_i T^3} \tag{33}$$

$$\psi_i \;\to\; \psi_i + \Delta_{i-1} - \Delta_i \tag{34}$$

Since the $\mathbf{t}_i$ remain intact under (32), the gauge transformation of (θ_i, ψ_i) has no effect on the geometry of the discrete string.

A priori, the fundamental range of the bond angle is $\theta_i \in [0, \pi]$ while for the torsion angle the range is $\psi_i \in [-\pi, \pi)$. Thus we identify (θ_i, ψ_i) as the canonical latitude and longitude angles of a two-sphere $\mathbb{S}^2$. For practical purposes we find it useful to extend the range of θ_i into negative values $\theta_i \in [-\pi, \pi] \; mod(2\pi)$. We compensate for this two-fold covering of $\mathbb{S}^2$ by a $\mathbb{Z}_2$ symmetry which takes the following form:

$$\begin{aligned} \theta_k &\to -\,\theta_k \qquad \text{for all} \;\; k \geq i \\ \psi_i &\to \quad \psi_i - \pi \end{aligned} \tag{35}$$

This is a special case of (33), (34), with

$$\begin{aligned} \Delta_k &= \pi \qquad \text{for} \;\; k \geq i+1 \\ \Delta_k &= 0 \qquad \text{for} \;\; k < i+1 \end{aligned}$$

C. Generalized discrete Kirchhoff energy and solitons

The energy function used in the article is obtained by a direct naive discretization of (19), and by replacing curvature and torsion by the discrete bond and torsion angles [2, 4, 9] . In particular, we use

$$(\partial_s \kappa)^2 \;\to\; (\theta_{i+1} - \theta_i)^2$$

Thus,

$$(\partial_s \kappa)^2 + \lambda\,(\kappa^2 - m^2)^2 \;+\; \frac{d}{2}\kappa^2\tau^2 \;-\; b\kappa^2\tau \;-\; a\tau \;+\; \frac{c}{2}\tau^2$$

becomes

$$\sum_{i=1}^{n} \left\{ -2\theta_{i+1}\theta_i + 2\theta_i^2 + \lambda\,(\theta_i^2 - m^2)^2 + \frac{d}{2}\,\theta_i^2\phi_i^2 - b\,\theta_i^2\phi_i - a\,\phi_i + \frac{c}{2}\phi_i^2 \right\} \tag{36}$$

which is the (θ, ψ) contribution to the energy function in Eqn. (4) of the article.

The conventional discrete NLS equation is known to support solitons [10]. Thus we expect that (36) supports soliton solutions as well: We follow (22) to eliminate the torsion angle,

$$\psi_i[\theta] \;=\; \frac{a + b\theta_i^2}{c + d\theta_i^2} \;=\; a\frac{1 + (b/a)\theta_i^2}{c + d\theta_i^2} \tag{37}$$

For bond angles we then have

$$\theta_{i+1} = 2\theta_i - \theta_{i-1} + \frac{dV[\theta]}{d\theta_i^2}\theta_i \qquad (i = 1, ..., N) \tag{38}$$

We set $\theta_0 \;=\; \theta_{N+1} \;=\; 0$, and $V[\theta]$ is given by (24). To solve this numerically, we use the iterative equation [2, 11]

$$\theta_i^{(n+1)} = \theta_i^{(n)} - \epsilon \left\{ \theta_i^{(n)} V'[\theta_i^{(n)}] - (\theta_{i+1}^{(n)} - 2\theta_i^{(n)} + \theta_{i-1}^{(n)}) \right\} \tag{39}$$

where $\{\theta_i^{(n)}\}_{i\in N}$ is the nth iteration of an initial configuration $\{\theta_i^{(0)}\}_{i\in N}$ and ϵ is some sufficiently small but otherwise arbitrary numerical constant. We choose $\epsilon \;=\; 0.01$, in our simulations. The fixed point of (39) is independent of the value of ϵ, and clearly a solution of (38).

Once the fixed point is found, the corresponding torsion angles are obtained from (37). The frames are then constructed from (30), and the entire chain is constructed using (31).

We do not know of an analytical expression of the soliton solution to the equation (38). But an *excellent* approximative solution can be obtained by discretizing the topological soliton (21) [2]:

$$\theta_i \;\approx\; \frac{\mu_1 \cdot e^{\gamma_1 (i-s)} - \mu_2 \cdot e^{-\gamma_2 (i-s)}}{e^{\gamma_1 (i-s)} + e^{-\gamma_2 (i-s)}} \tag{40}$$

Here $(\gamma_1, \gamma_2, \mu_1, \mu_2, s)$ are parameters. The μ_1 and μ_2 specify the asymptotic θ_i-values of the soliton. Thus, these parameters are entirely determined by the character of the regular, constant bond and torsion angle structures that are adjacent to the soliton. In particular, these parameters are not specific to the soliton *per se*, but to the adjoining regular structures. The parameter s defines the location of the soliton along the string. This leaves us with only two loop specific parameter, the γ_1 and γ_2. These parameters quantify the length of the bond angle profile that describes the soliton.

For the torsion angle, (37) involves one parameter (a) that we have factored out as the overall relative scale between the bond angle and torsion angle contributions to the energy. This parameter determines the relative flexibility of the torsion angles, with respect to the bond angles. Then, there are three additional parameters $(b/a, c/a, d/a)$ in the remainder $\psi[\theta]$. Two of these are again determined by the character of the regular structures that are adjacent to the soliton. As such, these parameters are not specific to the soliton. The remaining single parameter specifies the size of the regime where the torsion angle fluctuates.

On the regions adjacent to a soliton, we have constant values of (θ_i, ψ_i). In the case of a protein, these are the regions that correspond to the standard regular secondary structures. For example, the standard right-handed α-helix is obtained by setting

$$\alpha - \text{helix}: \quad \begin{cases} \theta \approx \frac{\pi}{2} \\ \psi \approx 1 \end{cases} \tag{41}$$

and for the standard β-strand

$$\beta - \text{strand}: \quad \begin{cases} \theta \approx 1 \\ \psi \approx \pi \end{cases} \tag{42}$$

All the other standard regular secondary structures of proteins such as 3/10 helices, left-handed helices *etc.* are similarly modeled by definite constant values of θ_i and ψ_i. Protein loops correspond to solitons, the regions where the values of (θ_i, ψ_i) are variable.

The presence of solitons *significantly* reduces the number of parameters in (36), increasing the predictive power. In particular, the number of parameters is far smaller than the number of amino acids, along the protein backbone.

III. MYOGLOBIN MULTISOLITON

To construct the multisoliton solution of (38), (37) that models the Cα backbone of the crystallographic myoglobin structure with Protein Data Bank code 1ABS in the article, we use a combination of the `GaugeIT` and `Propro` packages, described at

$$\texttt{http://folding-protein.org/}$$

The analysis starts with the inspection of the bond and torsion angle spectrum with the help of the $\mathbb{Z}_2$ symmetry (35), to identify the individual solitons. In Figure 1 we show the (θ_i, π_i) spectrum both for 1ABS, and for the multisoliton we have constructed; the Cα RMS distance between the two is around 0.8 Å. In Table I we show the parameter values that we have found. There are 92 parameters that describe the 154 different amino acids.

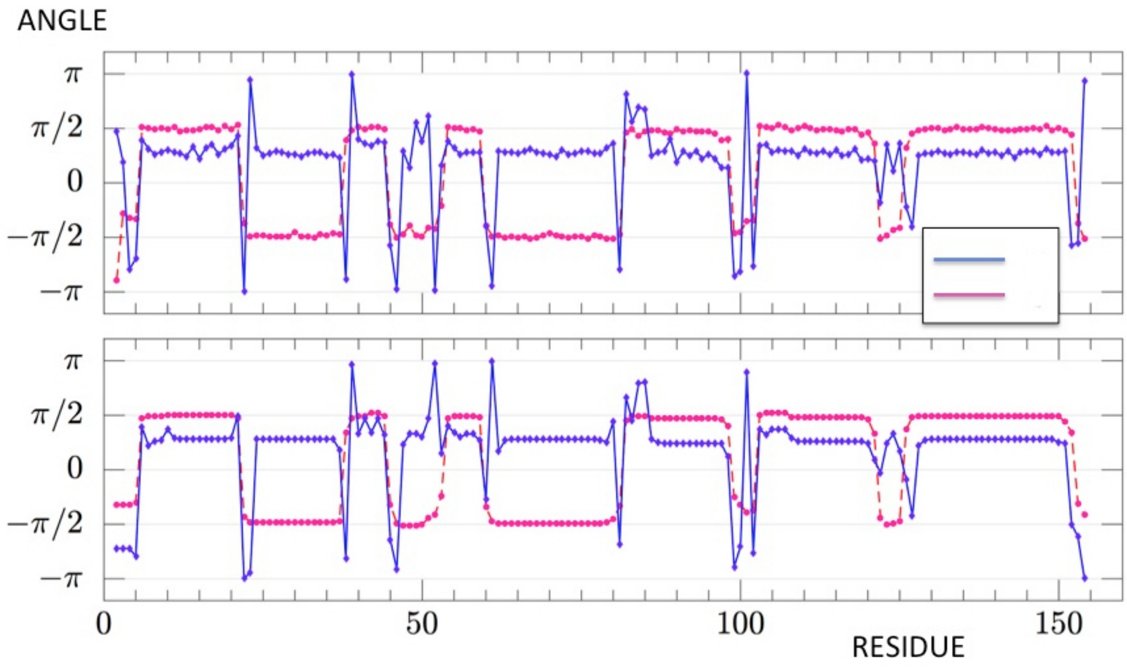

FIG. 1: *Color online: Top:* The bond (θ) and torsion (ψ) angle spectrum of the PDB structure 1ABS. *Bottom:* The bond (θ) and torsion (π) angle spectrum of the multisoliton. Note that the angles are defined modulo 2π.

Finally, we comment on the various versions of the Gõ model [13]. These approaches have played a very important rôle, to gain insight to protein folding in particular when the power

number	start site	end site	center site	d/2	λ_1	λ_2	a	c/2	b	m_1	m_2
1	1	8	4	6.283059e-08	12.078732	3.9176859	-3.107281e-08	4.193225e-08	2.170112e-06	1.01168	1.542089
2	9	22	20	1.00696e-08	3.436446	2.029519	-7.237373e-08	1.250037e-08	-1.080896e-06	1.579765	1.513911
3	23	40	36	1.849663e-09	7.320744	0.814552	-1.01612e-07	2.7051e-10	4.827056e-08	1.506042	1.542905
4	41	49	43	2.877262e-09	2.137929	0.657065	-9.048445e-08	2.550927e-11	1.202453e-06	1.655888	1.602375
5	50	54	52	3.837542e-09	0.885448	5.970876	-2.340973e-07	1.181006e-08	-3.301817e-07	1.363863	1.536391
6	55	78	58	2.436747e-09	8.707656	0.8339	-9.640594e-08	5.113017e-11	4.77647e-07	1.549945	1.536618
7	79	84	80	9.507981e-15	0.973448	2.140467	-7.400912e-09	3.471722e-10	3.834484e-09	1.462046	1.546792
8	85	99	97	2.725827e-14	1.32568	2.911392	-1.375057e-13	1.745762e-14	5.605584e-13	1.477527	1.020112
9	100	106	102	6.128919e-09	10.480469	4.242685	-1.213871e-07	4.957782e-11	1.371842e-06	1.222433	1.653224
10	107	122	120	3.911725e-08	0.800539	1.289546	-2.035177e-07	7.300473e-12	1.135981e-06	1.514903	1.602549
11	123	149	124	3.868921e-09	3.1520826	0.914394	-1.077984e-07	3.749198e-11	1.027845e-06	1.557863	1.551461
12	150	154	151	5.692258e-09	1.012151	1.06336	-1.117553e-07	2.192283e-10	8.620106e-07	1.400077	1.328203

TABLE I: The parameters in the energy function for 1ABS

of computers is insufficient for any kind of serious all-atom folding simulations. In these models the folded configuration is presumed to be known; the individual atomic coordinates of the folded protein chain appear as an input. A simple energy function is then introduced, tailored to ensure that the known folded configuration is a minimum energy ground state; the energy could be as simple as a square well potential which is centered at the native conformation.

Since the positions of all the relevant atoms appear as parameters in these models, they contain more parameters than unknown and thus no predictions can be made. Only a description is possible. From the point of view of a system of equations, these models are over-determined. In any *predictive* energy function the number of adjustable parameters must remain *smaller* than the number of independent atomic coordinates.

[1] M. Spivak, *A Comprehensive Introduction to Differential Geometry* (Five Volumes) 3rd ed. (Publish or Perish, Inc. Berkeley, CA, U.S.A., 1999)

[2] A.J. Niemi, in C. Chamon, M.O. Goerbig, R. Moessner, L.F. Cugliandolo (Eds.) *Topological Aspects of Condensed Matter Physics: Lecture Notes of the Les Houches Summer School* Vol. 103 (Oxford University Press, Oxford, 2017)

[3] Hu, S., Jiang, Y. & Niemi, A.J., *Phys. Rev.* **D87** 105011 (2013)

[4] Ioannidou, T., Jiang, Y., & Niemi, A.J., *Phys. Rev.* **D90** 025012 (2014)

[5] Dill, E.H., *Arch. Hist. Ex. Sci.* **44** 1(1992)

[6] Langer, J., Singer, D., *SIAM Rev.* **38** 605 (1996)

[7] L.A. Takhtadzhyan, L.D. Faddeev, *Hamiltonian approach to soliton theory* (Springer Verlag, Berlin, 1987)

[8] N. Manton and P. Sutcliffe, *Topological Solitons* (Cam- bridge University Press, Cambridge, 2004)

[9] Hu, S., Lundgren, M. & Niemi, A.J., *Phys. Rev.* **E83** 061908 (2011)

[10] P.G. Kevrekidis, *The discrete Nonlinear Schödinger equation: Mathematical Analysis, Numerical Computations and Physical Perspectives* (Springer Verlag, Berlin, 2009)

[11] Molkenthin, N., Hu, S. & Niemi, A.J., *Phys. Rev. Lett.* **106** 078102 (2011)

[12] Peng, X., Sieradzan, A. & Niemi, A.J., *Phys. Rev* **E94** 062405 (2016)

[13] Gō, N. N., *Annual Review of Biophysics and Bioengineering* **12** 183(1983)
